# 1495. Mpox Vaccine Immunity in People Living with HIV (PLWH) During The 2022 Mpox Outbreak in New York City

**DOI:** 10.1093/ofid/ofad500.1330

**Published:** 2023-11-27

**Authors:** Angelica C Kottkamp, Aaron Oom, Kesi K Wilson, Stephanie Rettig, Olivia Frank, Celia Engelson, Tamia S Davis, Irma Noriega, Jacqueline Callahan, Samantha Yip, Heekoung Youn, Julia Wagner, Ellie Carmody, Lalitha Parameswaran, Michael Tuen, Jimmy P Wilson, Shelby J Goins, Lisa Zhao, Samuel Nweke, Sarah Haiken, Pamela Suman, Amanda Dontino, Jennifer Rosen, Jane R Zucker, Ralf Duerr, Marie I Samanovic, Mark J Mulligan

**Affiliations:** NYU Grossman School of Medicine, New York, NY; New York University Grossman School of Medicine, New York, New York; NYU Grossman School of Medicine, New York, NY; NYU Langone Vaccine Center, New York, New York; NYU Langone, new york, New York; NYU Vaccine Research Center, New York, New York; NYU Langone Health Vaccine Center, Yonkers, New York; NYU Langone Medical Center, NY, New York; NYU Langone Health Vaccine Center, Yonkers, New York; NYU Grossman School of Medicine, New York, NY; Nyusim, New York, New York; NYU Grossman School of Medicine, New York, NY; NYU Langone Health, BROOKLYN, New York; NYU Langone Health, NYU Langone Vaccine Center, New York, New York; NYU Langone Health, BROOKLYN, New York; NYU Langone, new york, New York; NYU Langone, new york, New York; NYU Langone Health, BROOKLYN, New York; NYU Langone Health, BROOKLYN, New York; NYU Langone Health, BROOKLYN, New York; NYU Langone, new york, New York; NYU Langone Vaccine Center, New York, New York; NYC Department of Health and Mental Hygiene, Long Island City, New York; NYC Department of Health and Mental Hygiene, Long Island City, New York; NYU Langone Health, BROOKLYN, New York; NYU Grossman School of Medicine, New York, NY; NYU Grossman School of Medicine, New York, NY

## Abstract

**Background:**

The WHO estimated that in 2022 49% of people affected by Mpox were PLWH. Due to initial vaccine shortage in 2022, a 1/5 dose of vaccine given intradermally (ID) was FDA-authorized (8/9/22). The ID regimen had never been evaluated in PLWH. Here we describe the characteristics, and IgG responses of Mpox vaccination in PLWH and HIV-negative participants.

**Figure:**
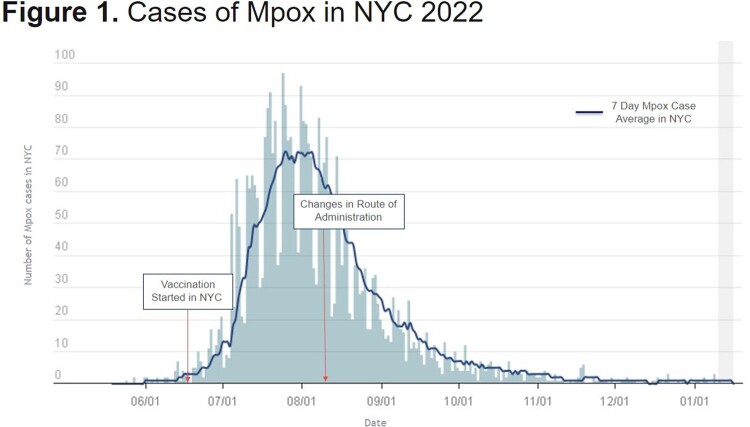

**Methods:**

We are conducting a longitudinal, observational study of adults with and without HIV who received the JYNNEOS vaccine or had Mpox infection in NYC. Clinical, demographic information, and Mpox H3L-specific serum IgG titers are assessed.

**Figure:**
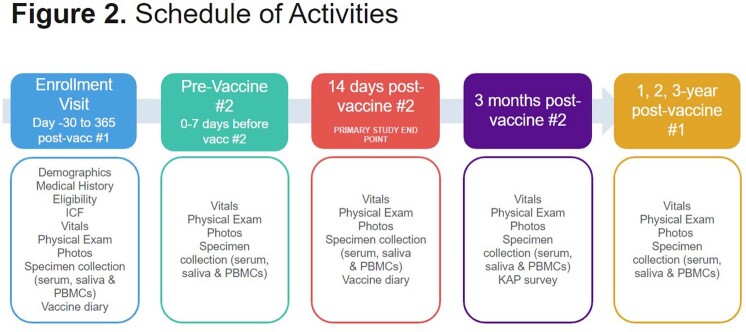

**Results:**

145 participants are enrolled: 25% are PLWH (median CD4 685 cells/mm3), 14% had Mpox disease and 21% had prior smallpox vaccination. 71% with Mpox disease were PLWH. The cohort is racially/ethnically diverse, mostly men (81%) and LGBTQ+ (90%). A third of the participants were recruited from NYC DOHMH pop-up vaccination site (34%), followed by peer or professional referral (20% & 19%). Routes of vaccination were: SC-ID (49%), ID-ID (21%), SC-SC (20%), and ID-SC (10%). Redness (37%) & swelling (21%) at site of injection were common reactions. Prior smallpox vaccination was associated with higher and sustained IgG titers even after one vaccine dose. Excluding such participants, antibody titers start declining post-dose 2 (antibody half-life of 103 days). There was no difference in IgG titers at 3 months post-dose 2 between PLWH and HIV-negative for ID or any other route. Titers at 3 months post-dose 2 in PLWH correlated with CD4 counts. Longer interval between the two doses contributed to higher titers. Mpox infection yielded to significantly higher titers than vaccination post-dose 1, but no after 2 doses.

**Figure:**
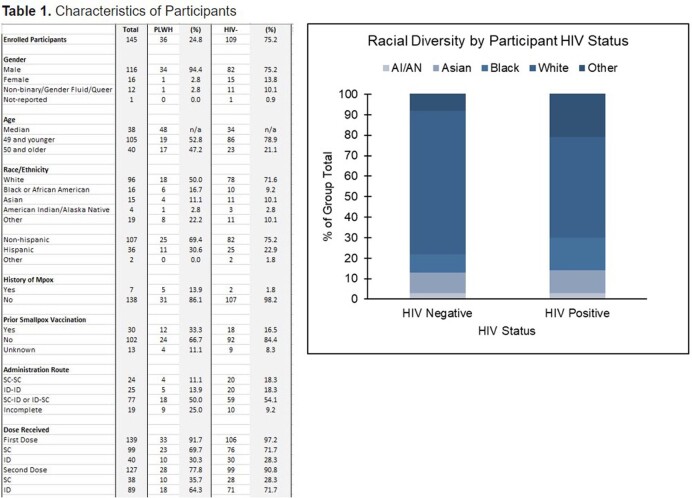

**Figure:**
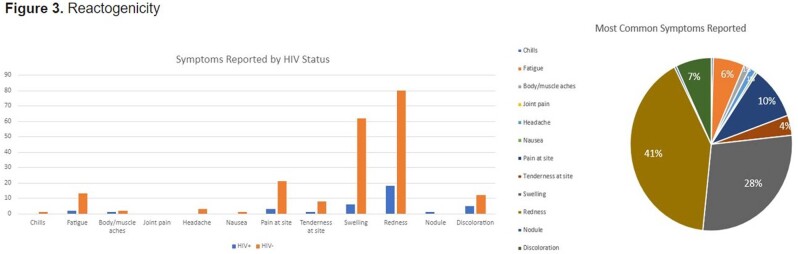

**Figure:**
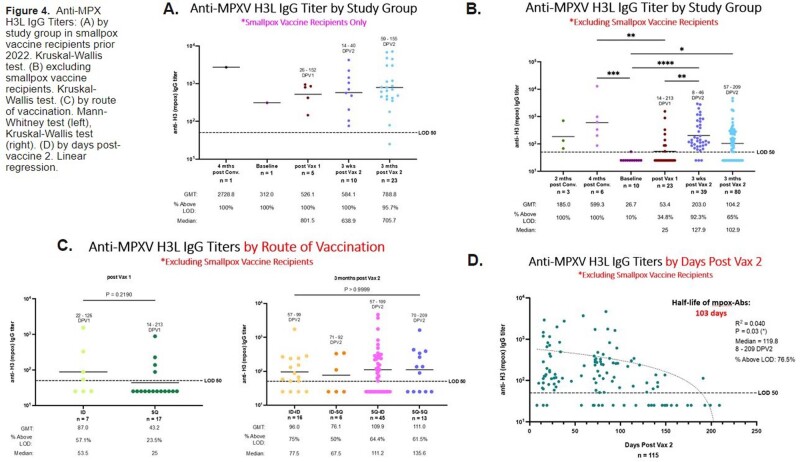

**Conclusion:**

Our study highlights the disproportionate burden of Mpox infection on PLWH. A diverse population representative of those most affected by 2022 Mpox was recruited. The vaccine, whether ID or SC, was well tolerated in PLWH and HIV-negative. More than two-thirds of participants with Mpox disease were PLWH. Individuals with prior smallpox vaccination maintain strong antibody levels post-dose 2, however, levels decline in individuals with no anterior prime. Our preliminary results show no negative impact of the ID dose regimen on antibody levels in PLWH.

**Figure:**
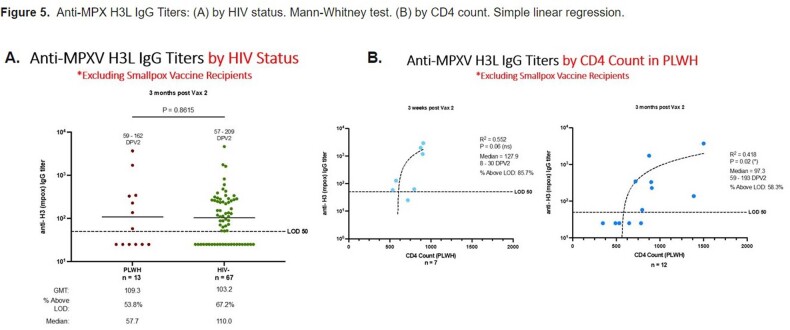

**Disclosures:**

**Ellie Carmody, MD, MPH**, AstraZeneca: Stocks/Bonds|Merck: Stocks/Bonds **Lalitha Parameswaran, MD, MPH**, Pfizer: Grant/Research Support **Mark J. Mulligan, M.D.**, Lilly: Grant/Research Support|Meissa Vaccines, Inc.: Advisor/Consultant|Meissa Vaccines, Inc.: Board Member|Merck: Advisor/Consultant|Merck: Board Member|Pfizer: Advisor/Consultant|Pfizer: Board Member|Pfizer: Grant/Research Support|Sanofi: Grant/Research Support

